# Utility of High-Throughput Genomic Analysis for Genetic Counseling in Large Family with Wilson Disease Carrying a Novel 28-bp *ATP7B* Splice-Junction Deletion

**DOI:** 10.3390/diagnostics16172682

**Published:** 2026-08-22

**Authors:** Areerat Hnoonual, Dhipsukon Pongborriboon, Nattaphon Wansom, Noppadol Kietsiriroje, Oradawan Plong-On, Pornprot Limprasert

**Affiliations:** 1 Division of Molecular Pathology, Department of Pathology, Faculty of Medicine, Prince of Songkla University, Songkhla 90110, Thailand; areerat.h@psu.ac.th (A.H.); oradawan@hotmail.com (O.P.-O.); 2 Genomic Medicine Center, Songklanagarind Hospital, Prince of Songkla University, Songkhla 90110, Thailand; 3 Department of Medicine, Faculty of Medicine, Prince of Songkla University, Songkhla 90110, Thailand; dhipsukon.pon@gmail.com (D.P.); noppadol.k@psu.ac.th (N.K.); 4 Department Internal Medicine, Yantakao Hospital, Trang 92140, Thailand; nattaphon9655@gmail.com

**Keywords:** *ATP7B*, deletion, genetic counseling, splice-junction deletion, Wilson disease

## Abstract

**Background/Objectives:** Wilson disease (WD) is an autosomal recessive disorder of copper metabolism caused by pathogenic variants in the *ATP7B* gene. Early diagnosis and appropriate treatment are essential for preventing irreversible complications. This study demonstrated the clinical utility of integrated high-throughput genomic analysis for molecular diagnosis and genetic counseling in a large Thai family affected by WD. **Methods:** A 32-year-old woman with clinical features suggestive of WD underwent clinical, biochemical, and molecular genetic evaluations, including sequencing of the entire *ATP7B* gene and SNP microarray. Fluorescent PCR followed by capillary electrophoresis was used for segregation analysis in available family members. SNP microarray analysis and whole-exome sequencing were performed on the proband’s husband to identify pathogenic variants in the *ATP7B* gene and other disease-associated genes for reproductive risk assessment. **Results:** The proband presented with hepatic dysfunction, Kayser–Fleischer rings, low serum ceruloplasmin, and a family history of fatal liver disease. She also developed progressive weakness, with nerve conduction findings consistent with axonal sensorimotor polyneuropathy predominantly affecting the lower limbs. Sequencing identified a novel homozygous 28-bp splice-junction deletion, c.4022-24_4025del, which disrupted the canonical splice acceptor site at the intron 19/exon 20 boundary and was classified as pathogenic variant. Segregation analysis confirmed carrier status in the proband’s father and identified heterozygous carrier or homozygous wild-type status among her living siblings. SNP microarray analysis revealed a 46.7 Mb copy-neutral long contiguous stretch of homozygosity (CN-LCSH) encompassing *ATP7B*, with CN-LCSH regions accounting for 2.046% of the total autosomal genome. These findings potentially reflected segmental uniparental isodisomy or identity by descent, while the overall homozygosity pattern did not support recent consanguinity. Combined genomic analyses of the proband’s husband revealed no pathogenic or likely pathogenic *ATP7B* variants. Based on the available testing, all offspring are expected to be heterozygous carriers, and the risk of an affected child is considered very low. **Conclusions:** This study highlights the value of integrated genomic analysis for molecular diagnosis, cascade testing, and reproductive risk counseling. Further functional studies should be conducted to validate their pathogenicity.

## 1. Introduction

Wilson disease (WD; OMIM #277900) is an autosomal recessive disorder of copper metabolism caused by loss-of-function pathogenic variants in the *ATP7B* gene on chromosome 13q14.3. ATP7B encodes a copper-transporting P-type ATPase (ATP7B protein) that transports copper into the trans-Golgi network for ceruloplasmin synthesis and mediates copper excretion into bile. Loss of ATP7B function results in systemic copper toxicity affecting multiple organs, primarily in the liver and central nervous system, as well as the cornea, kidneys, joints, and cardiac muscle, thereby contributing to the characteristic clinical features of WD [1]. Clinical manifestations vary widely and commonly include hepatic involvement ranging from chronic hepatitis and cirrhosis to acute liver failure, neurological deficits, psychiatric symptoms, and Kayser–Fleischer rings of the cornea [2].

The global prevalence of WD ranges from 1 in 10,000 to 1 in 30,000, with a carrier frequency of approximately 1 in 90 in the general population [3]. Higher prevalence has been reported in Asian countries and in populations with high rates of consanguinity [4,5]. A recent population-based study estimated the prevalence of WD in Thailand at 1 in 24,128 and the carrier frequency at 1 in 78 [6]. WD can present at any age, from early childhood to late adulthood, with reported onset ranging from 2 to nearly 70 years [7,8]. Diagnosis may be particularly challenging in pediatric patients, who often remain asymptomatic during the early stages of the disease. Early detection and intervention are critical for preventing irreversible complications and mortality [9,10]. However, the marked phenotypic heterogeneity of WD continues to pose a significant challenge for clinical diagnosis. Traditional diagnostic approaches rely on a combination of clinical findings, biochemical markers, and imaging studies; however, these strategies have inherent limitations [2,11]. Owing to the heterogeneous clinical presentation, only approximately 30% of patients with WD receive an accurate diagnosis at their initial medical consultation [12]. To improve diagnostic accuracy, a scoring system established at the 8th International Meeting on Wilson Disease in Leipzig incorporates clinical signs, histopathological findings, biochemical parameters, and genetic testing. A total score of ≥4 is considered diagnostic for WD, and genetic testing carries the highest weighting in this scoring system [2,11].

Genetic testing has therefore become an essential tool for confirming the diagnosis of WD, shortening the time to treatment, and facilitating accurate genetic counseling for at-risk family members. To date, more than 1000 variants in the *ATP7B* gene have been recorded in the Human Gene Mutation Database and ClinVar. The spectrum of *ATP7B* variants varies across geographic regions and ethnic populations [1,5,13]. The c.2333G>T (p.Arg778Leu) variant is the most commonly reported *ATP7B* variant in the Thai population and is also prevalent among Chinese, Korean, Japanese, and Taiwanese patients with WD [6,14,15,16,17,18,19,20,21], whereas c.3207C>A (p.His1069Gln) is the most frequently reported variant in European and North American populations [22,23]. Certain variants have also been associated with differences in clinical severity, highlighting the importance of molecular diagnosis for prognosis and long-term management. Missense variants represent the most common type of *ATP7B* variant, followed by nonsense, frameshift insertion/deletion, and splice-site variants. Pathogenic nonsense, frameshift, and splice-site variants in *ATP7B* frequently result in loss of protein function and are generally associated with more severe clinical phenotypes and earlier disease onset [1,24,25].

In this study, high-throughput genomic analysis was performed to evaluate a patient with suspected WD and her family members, leading to the identification of a novel homozygous 28-bp deletion in the *ATP7B* gene. Segregation analysis and genomic testing of the proband’s husband were subsequently conducted to support risk assessment for the patient’s offspring and other at-risk relatives. These findings highlight the value of integrated genomic analysis for molecular diagnosis, cascade testing, and reproductive counseling in families affected by WD.

## 2. Materials and Methods

### 2.1. Patient

**Figure 1:**
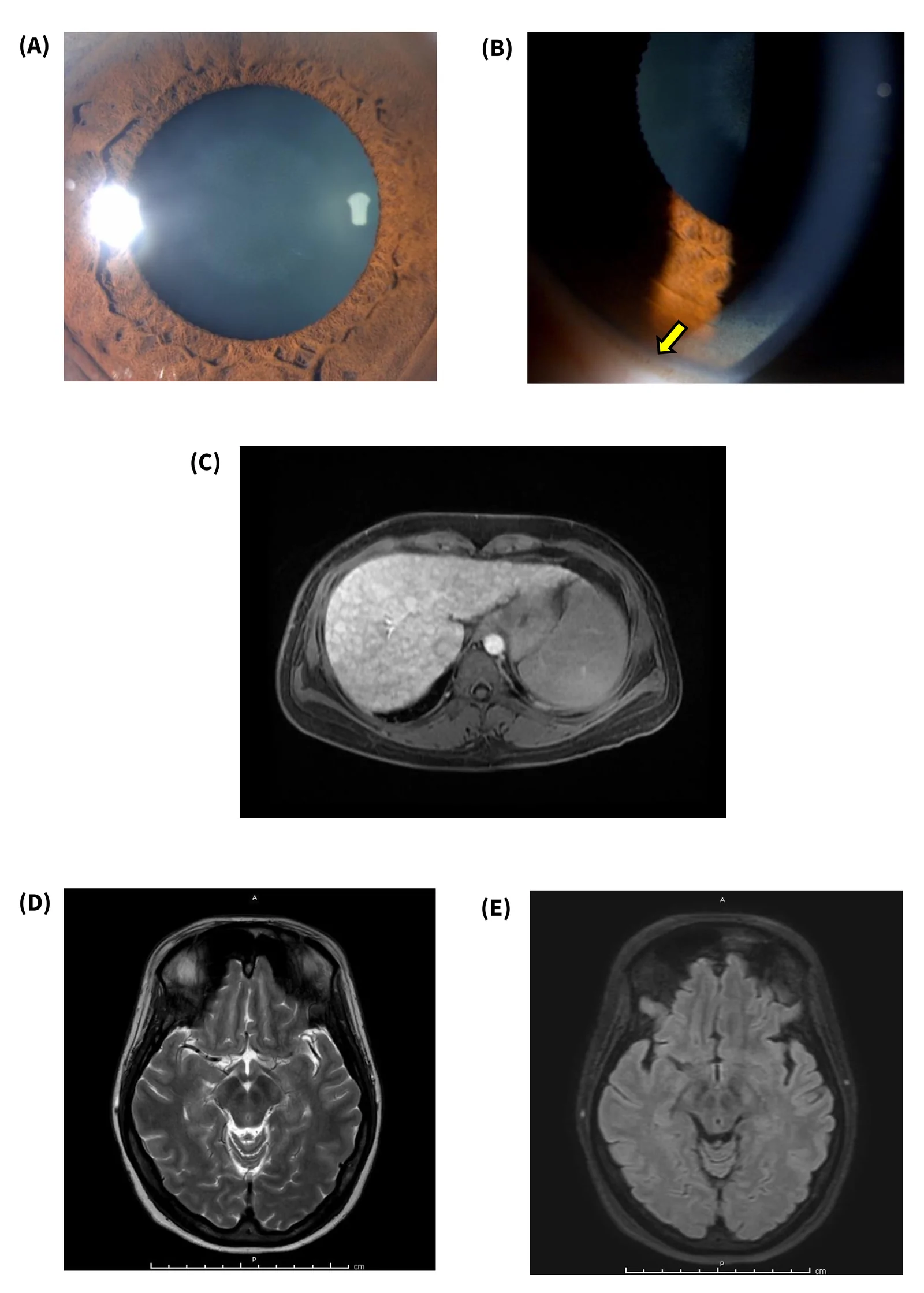
Significant clinical features of Wilson disease identified in the proband. (**A**) Sunflower cataract. (**B**) Copper deposition in the cornea (arrow). (**C**) Coarse liver parenchyma with a nodular hepatic surface, suspected cirrhosis, no arterial enhancing mass, and marked splenomegaly. (**D**) T2-weighted/fluid-attenuated inversion recovery image of the midbrain showing the “face of the giant panda” sign. (**E**) Bilateral symmetrical gradient-echo/susceptibility-weighted imaging hypointensities in the bilateral lentiform nuclei, prominent in the globus pallidi and dentate nuclei, with minimal hypointensities in the substantia nigra and red nuclei.

The proband was a 32-year-old woman with no known prior medical history who was referred to the hospital for suspected WD. She initially presented with jaundice and fatigue associated with a history of hemolytic anemia 10 months before admission. The reported episode had been evaluated at another hospital, and the original records and objective laboratory evidence of Coombs-negative hemolysis were unavailable. At presentation to our hospital, the patient had no anemia (hemoglobin, 12.1 g/dL; hematocrit, 36.6%); therefore, the historical episode was not included in the diagnostic score. One month before referral to our hospital, she had been admitted to a provincial hospital for evaluation of edema and suspected fluid accumulation. During that hospitalization, the patient developed progressive proximal muscle weakness that began in both lower limbs and subsequently extended to the upper limbs, accompanied by sensory loss in both legs and generalized areflexia. The progressive weakness impaired her gait and eventually rendered her bedbound for several months before the diagnosis of WD, although she remained able to perform basic activities of daily living. She later reported paresthesia in both feet, described as an electric shock sensation, which reduced sensation extending to the mid-thigh area, followed by numbness in all fingertips. She exhibited sunflower cataracts (Figure 1A) and Kayser–Fleischer rings in both eyes (Figure 1B). No psychiatric symptoms or movement disorders were observed. Despite no history of alcohol or substance abuse and negative viral hepatitis serology, her liver function progressively deteriorated. A notable family history of fatal liver disease was also documented. Neurological examination demonstrated intact cognitive function and normal cranial nerve findings, with variable motor strength across muscle groups. Sensory examination revealed reduced sensation in specific regions, and deep tendon reflexes were symmetrically absent in all extremities.

Laboratory investigations demonstrated severe hypoalbuminemia, and computed tomography of the abdomen showed findings consistent with liver cirrhosis (Figure 1C). Additional biochemical testing revealed a serum ceruloplasmin level of 8.4 mg/dL (0.084 g/L). Twenty-four-hour urinary copper excretion and quantitative hepatic copper concentration were not available. Brain magnetic resonance imaging (MRI) demonstrated bilateral symmetrical abnormalities in the basal ganglia (Figure 1D,E). Nerve conduction studies showed that compound muscle action potentials from the bilateral peroneal and tibial nerves and sensory nerve action potentials from the bilateral sural nerves could not be obtained. In the left upper limb, reduced motor response amplitudes were observed in the median (2.5 mV) and ulnar (5.0 mV) nerves, with normal distal motor latencies (4.1 and 3.0 ms) and normal conduction velocities (56.6 and 53.2 m/s, respectively). Sensory response amplitudes were normal at 29.5 µV in the left median nerve and 22.0 µV in the left ulnar nerve, with reduced conduction velocities of 46.2 and 43.1 m/s, respectively. These findings were consistent with symmetrical axonal sensorimotor polyneuropathy predominantly affecting the lower limbs (Supplementary Table S1).

The diagnosis of WD was established according to the scoring system provided by the EASL-ERN Clinical Practice Guidelines for WD [2,11]. The patient achieved a total score of 10, indicating a highly likely diagnosis of WD (Table 1).

### 2.2. Mutation Analysis

Genomic DNA was extracted from peripheral blood using the FlexiGene DNA Kit (QIAGEN, Hilden, Germany) according to the manufacturer’s protocol. In the proband, all 21 exons of the *ATP7B* gene were amplified by PCR using previously reported primers [14,26,27], followed by bidirectional Sanger sequencing. The detected variant was classified according to the ACMG-AMP standards and guidelines [28].

Fluorescent PCR was performed using DNA from all available family members to enable segregation analysis. PCR reactions were conducted in a total volume of 10 μL containing 50 ng of genomic DNA, 1.5 mM MgCl2, 0.2 mM of each dNTP, 0.3 μM fluorescently labeled forward primer (5′-6FAM-CTGTGGGCAAGATCCATTG-3′), 0.3 μM reverse primer (5′-TGCCACTGCAGCATTTGT-3′), 1× PCR buffer, and 0.5 units of Platinum™ Taq DNA Polymerase (Invitrogen, Thermo Fisher Scientific, Waltham, MA, USA). The PCR conditions comprised an initial denaturation at 95 °C for 2 min, followed by 35 cycles of denaturation at 95 °C for 30 s, annealing at 55 °C for 30 s, and extension at 72 °C for 30 s, with a final extension at 72 °C for 5 min. The amplified DNA fragments were separated by capillary electrophoresis using a 3500 Genetic Analyzer (Applied Biosystems, Foster City, CA, USA) and analyzed with GeneMapper Software version 5.0.

### 2.3. SNP Microarray

To determine regions of homozygosity associated with possible parental consanguinity, SNP microarray analysis was performed in the proband (II-17) and her husband (II-18) using the Human Infinium Global Screening Array with Cytogenetics-24 v1.0 BeadChip (Illumina, San Diego, CA, USA), which contains over 700,000 markers with a mean probe spacing of 4.0 kb. The results were analyzed using NxClinical 6.2 (BioDiscovery, LLC, El Segundo, CA, USA) based on Human Genome Build 38/hg38. The array was used to detect copy number gains and losses consistent with deletions and duplications in the genomic regions represented on the array, as well as long continuous stretches of homozygosity (LCSH), which may indicate loss of heterozygosity or uniparental isodisomy. The analysis focused on identifying potentially pathogenic regions of genomic imbalance containing ≥ 10 consecutive SNPs. The reporting cutoffs for gene-containing regions were 100 kb for deletions, 200 kb for duplications, and single or multiple LCSH regions > 10 Mb. Several databases were used for interpretation, including the Database of Genomic Variants, DECIPHER, ClinGen, ClinVar, the Thai CNV database, and an internal database.

### 2.4. Whole-Exome Sequencing

Whole-exome sequencing was performed in the proband’s husband to evaluate the presence of pathogenic variants in the *ATP7B* gene and other related genes, thereby facilitating accurate genetic counseling. Exome capture and library preparation were performed using the SureSelect Human All Exon V6+UTR Kit (Agilent Technologies, Santa Clara, CA, USA). Sequencing was performed on a NovaSeq 6000 platform (Illumina, San Diego, CA, USA). The assay covered the coding regions and approximately 10 bp of flanking intronic sequence adjacent to each exon. Sequencing reads were aligned to the human reference genome (GRCh37/hg19) using the Burrows–Wheeler Aligner, and variant calling was performed using the Genome Analysis Toolkit (GATK). Variants were annotated, filtered, and interpreted using VarSeq software (Golden Helix, Bozeman, MT, USA) according to the ACMG/AMP guidelines for sequence variant interpretation. Variant evaluation incorporated population frequencies, clinical database assertions, evolutionary conservation, and computational predictions of functional and splicing effects. The databases used for variant annotation included the 1000 Genomes Project, gnomAD, ClinVar, OMIM, dbSNP, NCBI RefSeq, and ExAC Gene Constraint. Computational prediction tools included VS-SIFT, VS-PolyPhen-2, PhyloP, GERP++, GeneSplicer, MaxEntScan, NNSplice, and PWM Splice Predictor.

The whole-exome sequencing data for the proband’s husband showed a mean depth of coverage of 36.9×, with 91.7% of target bases covered at ≥10×.

The study was conducted in accordance with the Declaration of Helsinki, and the protocol was approved by the Ethics Committee of the Faculty of Medicine, Prince of Songkla University (REC.66-353-14-4) on 25 August 2023. Informed consent for participation was obtained from all subjects involved in the study.

## 3. Results

**Figure 2:**
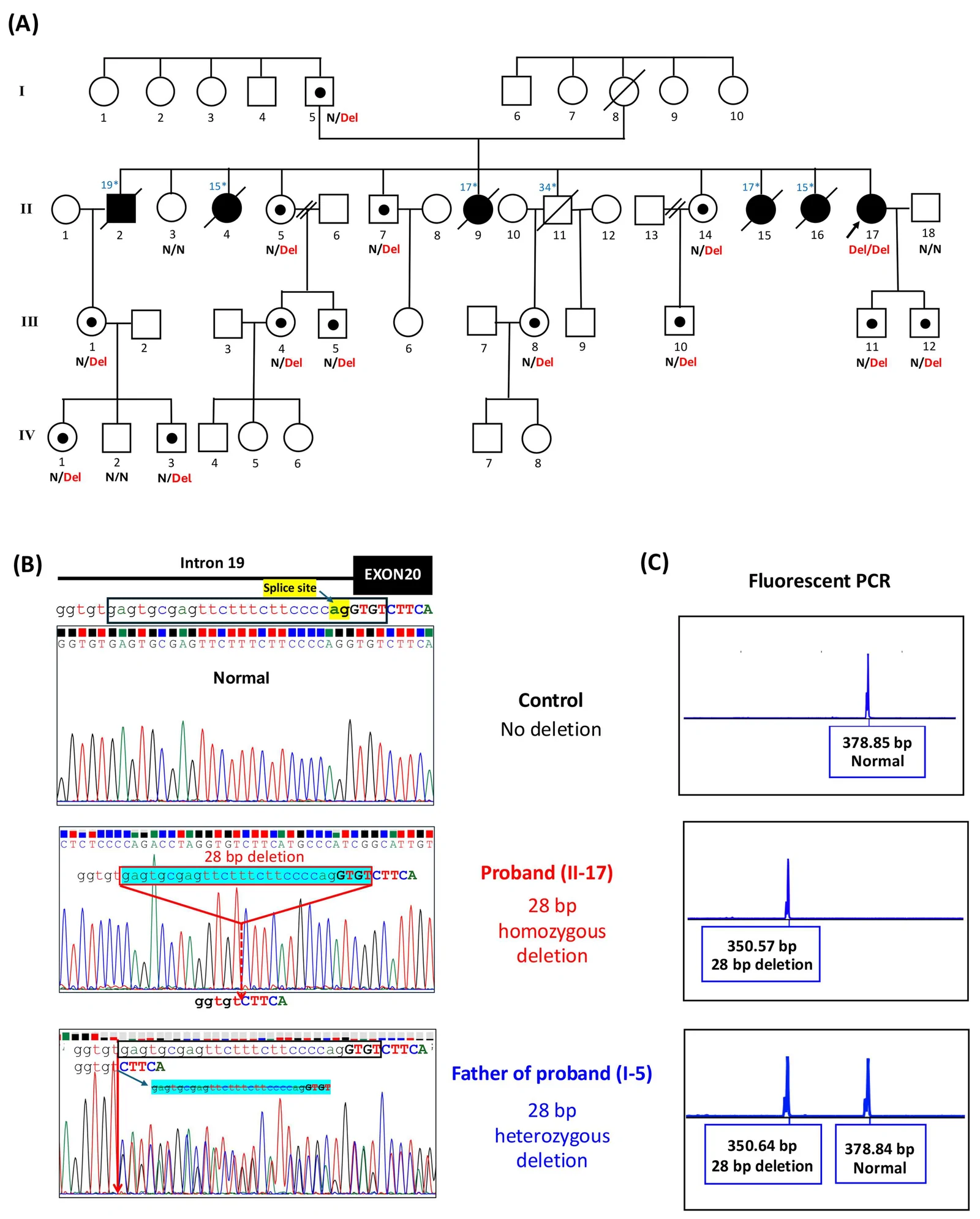
Novel *ATP7B* splice-junction deletion identified in this study. (**A**) Pedigree of the studied family. Circles represent females, and squares represent males. Filled symbols indicate individuals affected with or suspected of Wilson disease. Open symbols with a central dot indicate heterozygous carriers (N/Del), whereas open symbols without a dot indicate unaffected non-carriers (N/N). The proband (II-17) is indicated by a black arrow. “Del” denotes the novel splice-junction deletion c.4022-24_4025del, whereas “N” indicates the normal allele. An asterisk (*) indicates age at death. Individual II-11 was unaffected and died due to an accident. (**B**) Sanger sequencing identified the splice-junction deletion c.4022-24_4025del. The proband carried a homozygous deletion, and her father was heterozygous for the deletion. (**C**) Familial segregation analysis of the identified *ATP7B* deletion was performed using fluorescent PCR followed by capillary electrophoresis.

The clinical presentation and biochemical test results of the proband (Figure 2, II-17) raised strong clinical suspicion of WD. Therefore, sequencing of the entire *ATP7B* gene was performed and identified a novel homozygous splice-junction deletion, c.4022-24_4025del (NM_000053.4; chr13:g.52509834_52509861del), in the proband. This 28-bp deletion encompasses 24 nucleotides of intron 19 and 4 nucleotides of exon 20, including the canonical acceptor site (Figure 2 and Supplementary Figure S1). In silico analyses using MaxEntScan, SPiP, and SpliceAI predicted disruption of normal splicing, potentially resulting in a frameshift and premature termination codon. The variant was absent from the consulted population databases and had not previously been reported in individuals with *ATP7B*-associated disease. Considering the clinical manifestations, biochemical findings, and molecular analysis, the patient met the diagnostic criteria for WD according to the EASL–ERN Clinical Practice Guidelines. Based on its predicted loss-of-function effect, absence from population databases, and concordance with the patient’s phenotype, the variant met PVS1, PM2, and PP4 and was classified as pathogenic according to the ACMG/AMP variant interpretation guidelines (Supplementary Table S2).

Segregation analysis demonstrated that the patient’s father (I-5) was heterozygous for the c.4022-24_4025del variant, indicating carrier status. Maternal DNA was unavailable; therefore, maternal carrier status and biparental inheritance of the variant could not be confirmed. Familial segregation analysis using fluorescent PCR followed by capillary electrophoresis further identified either homozygous wild-type or heterozygous carrier genotypes among the patient’s living siblings. DNA samples from the deceased siblings were unavailable; therefore, their presumed homozygous genotypes were inferred from their clinical histories and the family segregation pattern and were not molecularly confirmed. Both sons of the proband (III-11 and III-12) were confirmed to be heterozygous carriers of the c.4022-24_4025del variant. Neither reported symptoms suggestive of WD at the time of genetic testing; therefore, no additional physical examination or biochemical evaluation was performed. To facilitate genetic counseling and reproductive risk assessment, whole-exome sequencing was subsequently performed on the proband’s husband (II-18). No pathogenic or likely pathogenic variants were identified in the *ATP7B* gene.

**Figure 3:**
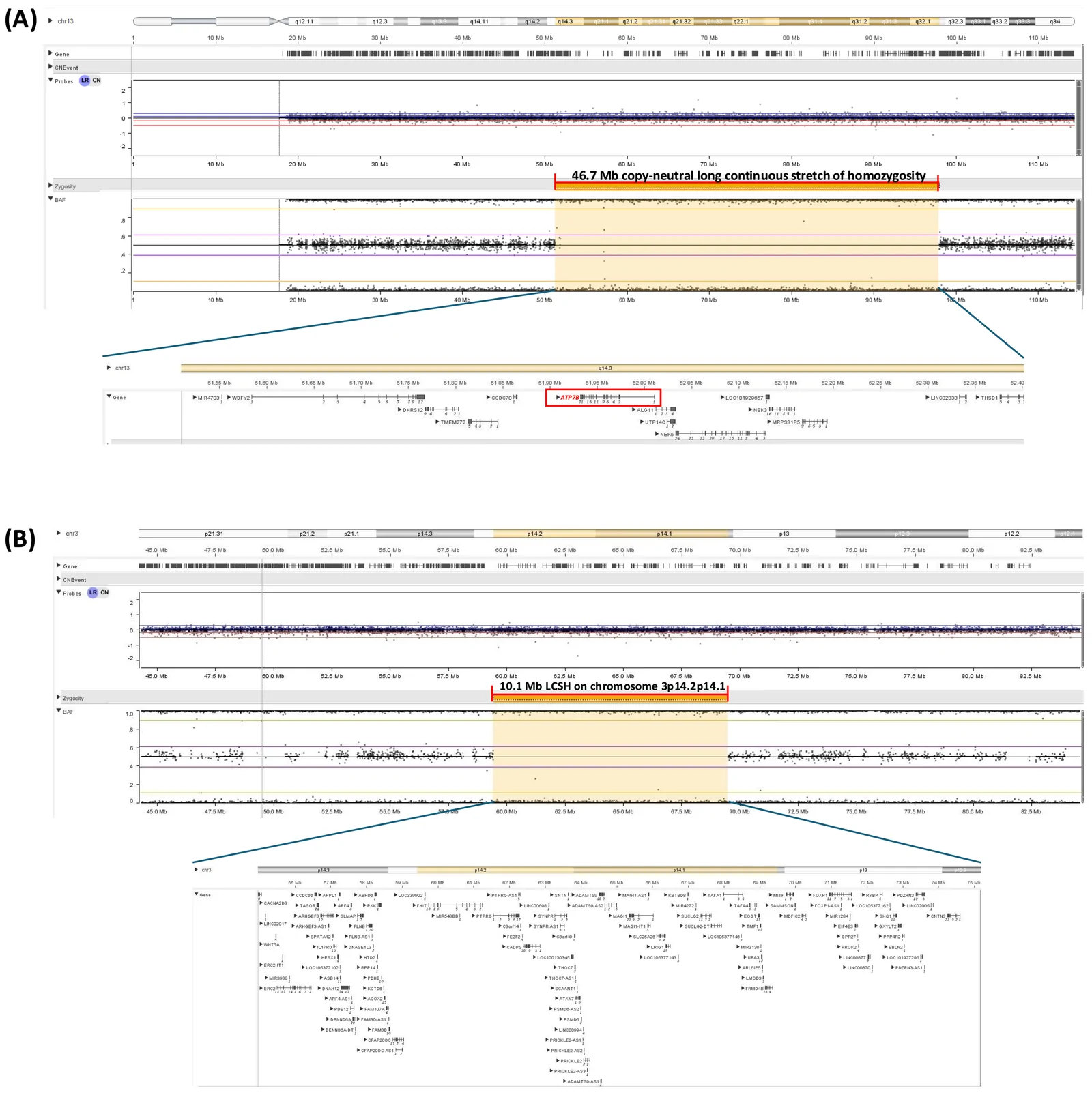
SNP microarray analysis results. (**A**) SNP microarray analysis of the proband with Wilson disease. SNP array analysis of the index case revealed a 46.7 Mb copy-neutral long continuous stretch of homozygosity (CN-LCSH) on the long arm of chromosome 13, spanning bands q14.3 to q32.2 from nucleotide 51,255,818 to 97,933,203. This large CN-LCSH contains the *ATP7B* gene among many other genes. (**B**) SNP array analysis of the patient’s husband showed a 10.1 Mb long continuous stretch of homozygosity on chromosome 3p14.2p14.1, which includes the OMIM disease-causing genes, *ATXN7* (*607640), *SLC25A26* (*611037), *EOGT* (*614789), and *LMOD3* (*616112).

SNP microarray analysis of the proband revealed a 46.7 Mb copy-neutral long continuous stretch of homozygosity (CN-LCSH) on chromosome 13q14.3q32.2 encompassed *ATP7B* and multiple other genes (Figure 3A and Supplementary Table S3). In this patient, the CN-LCSH regions accounted for 2.046% of total autosomal genome. Three additional regions of homozygosity measuring <10 Mb were also identified on chromosomes 3p21.31p21.2 (~5 Mb), 16p11.2q11.1 (~7.1 Mb), and Xq11.1q12 (~5.4 Mb). The presence of only one large homozygous region in the proband indicated that her parents were unlikely to be closely related. Therefore, the homozygous novel *ATP7B* variant identified in the proband and presumed in her affected deceased siblings was more consistent with distant shared ancestry or chance inheritance than with recent consanguinity.

In the proband’s husband, SNP array analysis revealed a normal male genotype without pathogenic copy number variations. However, a long continuous stretch of homozygosity spanning 10.1 Mb was identified on chromosome 3p14.2p14.1 (Figure 3B). Further whole-exome analysis of 48 genes located within this homozygous region, including the OMIM disease-causing genes *ATXN7*, *SLC25A26*, *EOGT*, and *LMOD3*, identified no pathogenic variants in the proband’s husband. Variants of uncertain significance detected in this individual are summarized in Supplementary Table S4.

## 4. Discussion

The present study identified a novel homozygous pathogenic 28-bp *ATP7B* splice-junction deletion, c.4022-24_4025del, in a Thai patient with WD, expanding the known spectrum of disease-causing *ATP7B* variants. The variant abolishes the canonical splice acceptor site at the intron 19/exon 20 boundary and is predicted to disrupt RNA splicing, potentially resulting in a frameshift and premature termination codon. Depending on the resulting transcript, this could lead to nonsense-mediated mRNA decay or production of a truncated protein. Loss-of-function variants of the *ATP7B* gene, particularly in a homozygous state, have previously been associated with more severe clinical phenotypes and earlier disease onset in WD [24,29]. The affected region is located within the C-terminal transmembrane region of ATP7B, encompassing transmembrane domains 7 and 8 (TM7 and TM8), which contribute to the copper translocation channel and regulation of intracellular protein trafficking [30,31]. Leu1373, located at the end of TM8, is required for protein stability and trans-Golgi network retention under low-copper conditions, whereas the trileucine motif at positions 1454–1456 mediates copper-stimulated retrograde trafficking [32]. The functional importance of TM8 is further supported by studies of the Met1356Val substitution in mouse *ATP7B*, which is equivalent to Met1359 in the human protein, a highly conserved residue within the intramembrane copper-binding site of TM8 [33]. The Met1356Val variant was expressed at normal levels in vitro but lacked measurable copper transport activity in direct copper uptake assays, and caused hepatic copper accumulation and liver disease in vivo [34,35]. This loss of transport activity is consistent with the predicted role of Met1359 as one of the three coordinating ligands that form the intramembrane copper-binding site in Cu-ATPases. Therefore, substitution of this conserved methionine residue is expected to reduce the affinity of ATP7B for copper, indicating that variants affecting the TM8 region may impair copper transport activity [33]. Moreover, previous studies have suggested that *ATP7B* variants involving exons 18–20, including mutations affecting the ATP hinge region, transmembrane domains 7–8, and C-terminal deletions, may be associated with hepatic and/or hematological manifestations rather than neurological presentations [36,37]. Accordingly, the novel *ATP7B* variant identified in the present study is predicted to affect the structure or stability of the C-terminal region and may impair copper transport. However, these proposed effects are based on computational prediction and comparison with previously characterized variants. The pathogenic classification was supported by the predicted loss-of-function effect, absence from population databases, and the highly specific WD phenotype; nevertheless, the precise effects on RNA splicing and protein expression remain to be established experimentally.

**Figure 4:**
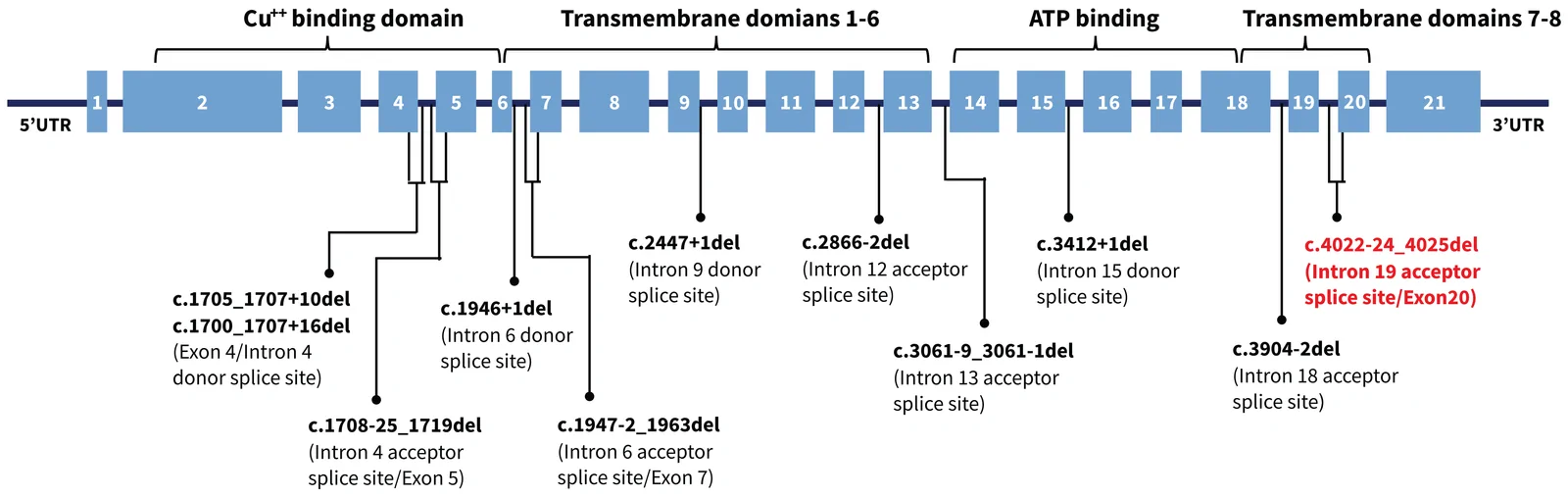
Distribution of pathogenic or likely pathogenic small splice-junction and canonical splice-site deletions (<50 bp) in the *ATP7B* gene reported in patients with Wilson disease worldwide. Variants are described according to the *ATP7B* reference transcript NM_000053.4, and genomic coordinates are based on the GRCh37/hg19 genome assembly. The novel c.4022-24_4025del variant (chr13:g.52509834_52509861del), which spans the intron 19/exon 20 junction and disrupts the canonical splice acceptor site of exon 20, is highlighted in bold red.

Although most reported pathogenic *ATP7B* variants are missense, nonsense, or small insertion/deletion variants, less common genetic mechanisms, including whole-exon deletions, deep intronic variants, promoter-region variants, and uniparental disomy, may complicate molecular diagnosis and genetic counseling [38]. Several deep intronic *ATP7B* variants have been functionally shown to cause aberrant pre-mRNA splicing [39], emphasizing the clinical relevance of evaluating variants beyond conventional coding regions. Splice-site variants may result in intron retention, exon skipping, or activation of cryptic splice sites, which may impair ATP7B protein function and contribute to the WD phenotype [40,41]. Among previously reported *ATP7B* splice-site variants, the well-characterized canonical splice-site variant c.1543+1G>C causes exon 3 skipping, altered protein interactions, loss of normal trans-Golgi network localization, and enhanced COMM domain-containing protein 1 (COMMD1)-mediated degradation of the mutant ATP7B protein via the ubiquitin–proteasome pathway [42]. The c.3244-2A>C variant similarly produces aberrant transcripts predicted to encode truncated ATP7B proteins. Patients carrying this splice-site variant presented with early disease onset, severe clinical manifestations, and poor prognosis. This suggested that careful evaluation of splice-altering variants may improve the accuracy of WD diagnosis and effectiveness of clinical genetic counseling [43]. The c.4022-24_4025del variant identified in the present study encompasses the canonical splice acceptor site at the intron 19/exon 20 junction and is located near the previously reported likely pathogenic c.4022-2A>C variant (ClinVar Variation ID: 553709). A larger deletion involving the same region, c.4021+87_4125-2del, which encompasses most of intron 19, the entire exon 20, and nearly all of intron 20, was identified in a patient with hepatic manifestations, disease onset at 10 years of age, and Kayser–Fleischer rings without neurological involvement. This deletion is predicted to produce an abnormal protein lacking TM8 and the C-terminal regulatory region [36,44]. Small splice-junction and splice-site deletions in *ATP7B* remain rarely reported in ClinVar (https://www.ncbi.nlm.nih.gov/clinvar, access on 26 May 2026) and are summarized in Figure 4. The splice-junction deletion identified in this study further expands the spectrum of rare *ATP7B* variants.

Clinical manifestations of WD are highly diverse. Although severe impairment of ATP7B function, particularly in patients carrying loss-of-function variants, may lead to more severe clinical phenotypes and earlier disease onset [24,29], genotype–phenotype correlations in WD remain inconsistent, and most previous studies have reported no clear correlation between *ATP7B* variants and clinical phenotype [45,46,47,48,49]. In the present study, the proband exhibited less severe clinical manifestations of WD than her siblings, who died before the age of 20 years, despite presumed homozygosity for the same variant. However, DNA samples and detailed clinical data from the deceased siblings were unavailable; therefore, their genotypes could not be confirmed, and the observed differences in clinical course cannot be attributed specifically to the c.4022-24_4025del variant. This difference may be explained by variable expressivity of WD within the same family [38]. Phenotypic discordance among family members sharing the same genotype has been reported in WD, including in studies of monozygotic twins and large multigenerational families, wherein differences in liver involvement, neuropsychiatric manifestations, and age at diagnosis have been observed [50,51,52,53,54]. Factors other than the primary disease-causing variant, including genetic modifiers, epigenetic factors, and environmental factors such as dietary copper intake, may contribute to clinical variability in WD [1,38,55].

During the diagnostic process, the patient also developed symptoms of peripheral neuropathy. Neurological manifestations of WD are heterogeneous and may involve both the central and peripheral nervous systems. Although movement disorders such as tremor, dystonia, and parkinsonism are considered the most characteristic neurological features, other manifestations have also been described, including epilepsy, autonomic dysfunction, sleep disturbances, and, less commonly, peripheral neuropathy [2,11,56]. Peripheral neuropathy in WD is relatively rare and remains poorly characterized, with limited reports describing its clinical presentation and significance [57]. Its underlying mechanism remains uncertain but may be multifactorial, involving direct copper-related neurotoxicity, metabolic disturbances, and impaired cellular energy metabolism [58,59,60]. In the present patient, the electrodiagnostic findings supported a symmetrical axonal sensorimotor polyneuropathy predominantly affecting the lower limbs. Advanced chronic liver disease and possible associated malnutrition or nutritional deficiencies may also have contributed to the peripheral neuropathy [61,62]. Given the progressive weakness, sensory loss, generalized areflexia, and axonal nerve conduction pattern, the possibility of concurrent Guillain–Barré syndrome, particularly the acute motor and sensory axonal neuropathy subtype, was also considered [63]. However, the patient showed no immediate response to intravenous immunoglobulin, and her neurological improvement occurred gradually during long-term follow-up. Cerebrospinal fluid analysis was not performed, and histopathological characterization by nerve biopsy was unavailable. Therefore, this possibility remained unconfirmed. The precise etiology of the peripheral neuropathy, including whether it was directly related to WD or the c.4022-24_4025del variant, could not be definitively established, and other contributing causes could not be excluded. In addition to D-penicillamine therapy for copper chelation, the patient received intensive rehabilitation and supportive care for polyneuropathy, followed by gradual improvement in her neurological symptoms. After several years of treatment, she returned to work and regained the ability to walk without a gait aid.

To identify family members at increased risk of carrying the c.4022-24_4025del variant in a heterozygous or homozygous state, targeted testing was subsequently performed in available relatives for WD screening and genetic counseling. Segregation analysis within the family further supported the pathogenicity of the identified variant, revealing heterozygous carrier status or homozygous wild-type status in the living siblings. Identification of carrier status in family members is clinically important for cascade screening and reproductive planning, particularly for offspring who may be at risk of inheriting the disease. Based on the identification of a homozygous variant in the patient, consanguinity was initially suspected because of the autosomal recessive inheritance pattern of the disease and reported occurrence of consanguineous marriage in some regions of Southern Thailand, where both parents shared the same ethnic background. Multiple copy-neutral LCSHs distributed throughout the genome generally represent autozygous segments that are identical by descent (IBD) from a common ancestor. These regions may harbor homozygous variants associated with autosomal recessive disorders. If genomic homozygosity is sufficiently extensive, it may raise suspicion of parental consanguinity. In contrast, one or more LCSHs restricted to a single chromosome raise the possibility of uniparental disomy [64,65]. In the present patient, a 46.7 Mb copy-neutral LCSH restricted to chromosome 13 encompassed *ATP7B* and was interpreted as likely segmental uniparental isodisomy (UPiD). Additional smaller LCSHs were also identified, resulting in total autosomal homozygosity of 2.046%. The father reported no known close relationship with his wife, and the overall LCSH distribution was not typical of the pattern of multiple large genome-wide LCSHs expected with recent consanguinity. Nevertheless, IBD from a distant common ancestor remained an alternative explanation [66]. SNP-based chromosomal microarray can detect copy-neutral LCSHs suggestive of uniparental disomy but cannot determine their parental origin without parental testing. Because maternal DNA was unavailable, the observed LCSH could not be definitively distinguished between segmental UPiD and IBD.

To enable more accurate recurrence risk assessment for WD in the proband’s offspring, genetic testing was extended to the patient’s husband. Because the proband carries a homozygous pathogenic *ATP7B* variant, all of their children are obligate heterozygous carriers of this variant. Therefore, genetic analysis of the patient’s husband was essential to determine whether he carried an additional pathogenic *ATP7B* variant that could increase the risk of WD in their sons through compound heterozygosity. Previous studies have suggested that WD-related symptoms in individuals carrying only one identified deleterious *ATP7B* variant may be explained by limitations of current genetic testing methods, which may fail to detect a second pathogenic variant, such as a large deletion or deep intronic variant [38,44,67,68]. This was unlikely in the present study because combined whole-exome sequencing and SNP microarray analyses were performed in the patient’s husband and demonstrated no pathogenic or likely pathogenic *ATP7B* variants. An additional SNP microarray finding revealed a long continuous stretch of homozygosity on chromosome 3p14.2p14.1 in the patient’s husband, suggesting inheritance from a distant common ancestor. To further investigate the clinical significance of this incidental finding, additional analysis of whole-exome sequencing data within this homozygous region was performed, although such analysis is not routinely conducted in general clinical practice. No pathogenic variants were identified within this region, suggesting no additional clinically significant finding for genetic counseling. Because the proband is homozygous for the c.4022-24_4025del variant and no pathogenic or likely pathogenic *ATP7B* variant was identified in her husband, all offspring are expected to be obligate heterozygous carriers. Therefore, the risk of an affected child is considered very low based on the available testing. Current comprehensive molecular testing of *ATP7B*, including sequencing of all coding exons and splice-site regions together with deletion/duplication analysis, can identify the genetic cause in approximately 98% of individuals with WD [69,70]. Nevertheless, no testing strategy can exclude all pathogenic variation, and a residual risk remains because WES and SNP microarray analysis may not reliably detect deep intronic or regulatory variants, variants in technically challenging or insufficiently covered regions, or small exon-level copy number variants. If clinically indicated, additional testing using whole-genome sequencing or long-read sequencing may improve the detection of such variants [38,71,72]. Rare *de novo* events or uniparental disomy also cannot be entirely excluded. These limitations should be considered during reproductive genetic counseling [1,38].

This study has some limitations. Maternal DNA was unavailable, precluding confirmation of maternal carrier status and biparental inheritance of the c.4022-24_4025del variant, as well as distinction between identity by descent and segmental uniparental isodisomy as the origin of the chromosome 13 CN-LCSH. In addition, transcript- and protein-level functional analyses were not performed; therefore, the predicted effects on RNA splicing and their potential consequences, including nonsense-mediated mRNA decay or production of a truncated protein, remain unconfirmed. Future studies using RT-PCR and cDNA sequencing of patient-derived RNA, minigene splicing assays, and functional expression studies in mammalian cell lines are warranted to establish the precise molecular consequences of this variant.

## 5. Conclusions

In this study, we identified a novel pathogenic 28-bp *ATP7B* splice-junction deletion, c.4022-24_4025del in a large family with WD. Integrated high-throughput genomic analyses, including whole-gene sequencing, SNP microarray analysis, and whole-exome sequencing performed in the proband and her husband, confirmed the molecular diagnosis in the family. These analyses also enabled more accurate reproductive risk assessment and genetic counseling for the family. Considering that WD is a treatable disorder, predictive genetic testing in asymptomatic at-risk individuals may facilitate early diagnosis and timely clinical management. These findings highlight the clinical value of comprehensive genomic analysis for improving molecular diagnosis, guiding cascade family screening, and informing genetic counseling in families with WD. Further functional studies at the RNA and protein levels are warranted to characterize the effects of the c.4022-24_4025del variant on RNA splicing and ATP7B protein function. Studies of additional patients and families are also needed to better define the phenotypic spectrum and genetic factors contributing to clinical variability in WD.

## Figures and Tables

**Table 1:** Clinical data of the proband according to the EASL-ERN Clinical Practice Guidelines [2].

Typical Clinical Symptoms and Signs		Score	Proband in This Study
Kayser–Fleischer rings	Present	2	Present (Score = 2)
	Absent	0	
Neuropsychiatric symptoms suggestive of Wilson’s disease (or typical brain MRI)	Present	2	Present (Score = 2)
	Absent	0	
Coombs-negative hemolytic anemia + high serum copper	Present	1	Absent (Score = 0)
	Absent	0	
Liver copper quantitative	>5× ULN (>250 µg/g)	2	Not available (liver biopsy was not done)
	<5× ULN (<250 µg/g)	1	
	Normal (<50 µg/g)	−1	
24 h urinary copper excretion (in the absence of acute hepatitis)	>2× ULN	2	Not available
	1–2× ULN	1	
	Normal	0	
	Normal, but >5× ULN after D-penicillamine test	2	
Serum ceruloplasmin	<0.1 g/L	2	0.084 g/L (Score = 2)
	0.1–0.2 g/L	1	
	Normal (>0.2 g/L)	0	
Rhodanine positive hepatocytes (only if quantitative copper measurement is not available)	Present	1	Not available
	Absent	0	
Disease-causing mutations detected	On both chromosomes detected	4	Detected on both chromosomes (Score = 4)
	On 1 chromosome detected	1	
	No mutations detected	0	
	**Total score**	**Score: 10** Highly likely *	

* ≥4 = highly likely, 2–3 = probable, 0–1 = unlikely; ULN, upper limit of normal.

## Data Availability

The original contributions presented in this study are included in the article and Supplementary Materials. Further inquiries can be directed to the corresponding author.

## Supplementary Materials

The following supporting information can be downloaded at: https://www.mdpi.com/article/10.3390/diagnostics16172682/s1, Figure S1: Electropherogram showing the homozygous splice-junction deletion, NM_000053.4(*ATP7B*):c.4022-24_4025del, identified in the proband with Wilson disease; Table S1: Electrodiagnostic report of the proband; Table S2: ACMG/AMP classification of the *ATP7B* c.4022-24_4025del variant; Table S3: Copy number variations and loss-of-heterozygosity regions identified in the proband with Wilson disease; Table S4: Variants of uncertain significance identified by whole-exome sequencing of the patient’s husband. References [2,11,28,73] are cited in the Supplementary Materials.
