# Supplementary material for: Utility of High-Throughput Genomic Analysis for Genetic Counseling in Large Family with Wilson Disease Carrying a Novel 28-bp ATP7B Splice-Junction Deletion
Source: Diagnostics (Basel). 2026 Aug 22;16(17):2682. doi: 10.3390/diagnostics16172682 (PMC13564501; doi:10.3390/diagnostics16172682)
Supplement: Supplementary file 1 [file diagnostics-16-02682-s001.zip › diagnostics-4430891-supplementary.pdf]

Supplementary data

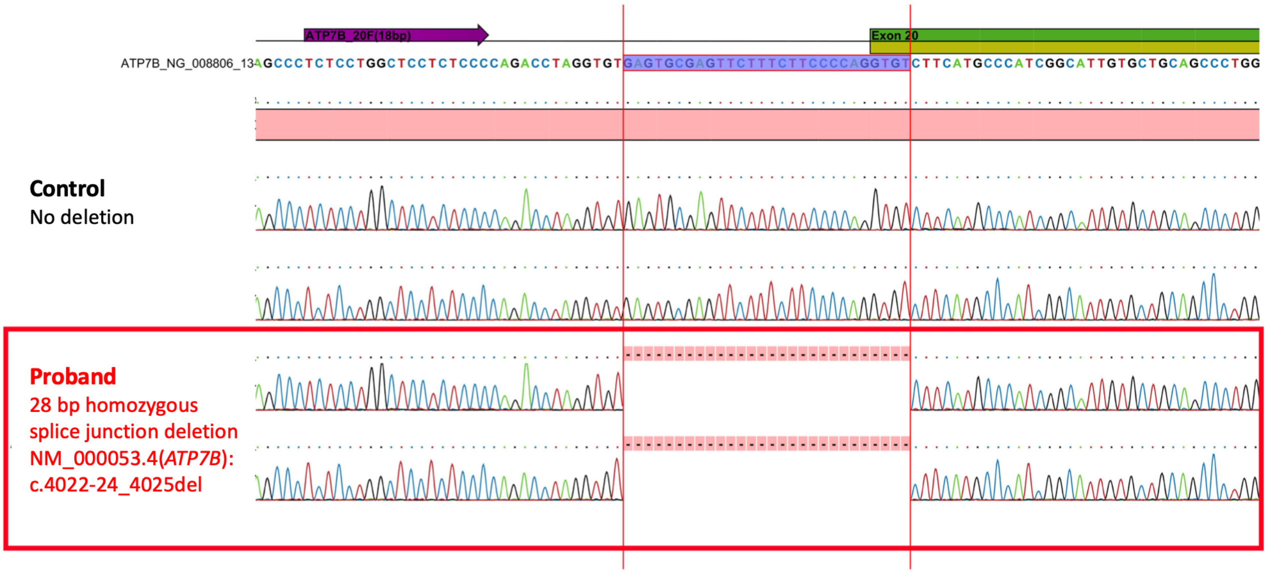

Figure S1. Electropherogram showing the homozygous splice-junction deletion, NM_000053.4(*ATP7B*):c.4022-24_4025del, identified in the proband with Wilson disease.

Table S1. Electrodiagnostic report of the proband

Motor nerve conduction study							
**Nerve/Site**	**Onset Lat (ms)**	**PF Amp (mV)**	**Duration (ms)**	**Area (mV·ms)**	**Distance (cm)**	**Lat diff (ms)**	**Velocity (m/s)**
**L Median – APB**							
Wrist	4.1	2.5	7.0	7.2	7	–	–
Elbow	8.1	2.1	6.5	6.9	23	4.1	56.6
**L Ulnar – ADM**							
Wrist	3.0	5.0	4.7	9.8	7	–	–
B Elbow	7.3	4.5	4.9	9.4	23	4.3	53.2
**L Peroneal – EDB**							
Ankle	NR	NR	NR	NR	8	–	–
**R Peroneal – EDB**							
Ankle	NR	NR	NR	NR	8	–	–
**L Tibial – AH**							
Ankle	NR	NR	NR	NR	8	–	–
**R Tibial – AH**							
Ankle	NR	NR	NR	NR	8	–	–

F-wave study			
**Nerve/Site**	**Min M Lat (ms)**	**Min F Lat (ms)**	**Min F-M Lat (ms)**
L Median – APB	4.1	27.3	22.9
L Ulnar – ADM	3.4	27.5	24.0

Sensory nerve conduction study						
**Nerve/Sites**	**Onset Lat (ms)**	**Peak Lat (ms)**	**Peak Amp (μV)**	**Duration (ms)**	**Distance (cm)**	**Velocity (m/s)**
**L Median – Digit II (Anti-dromic)**						
Wrist	2.8	3.7	29.5	2.7	13	46.2
**L Ulnar – Digit V (Anti-dromic)**						
Wrist	2.6	3.4	22.0	2.2	11	43.1
**L Sural – Ankle (A 7 cm, B 14 cm, C 21 cm)**						
Calf (A)	NR	NR	NR	NR	7	NR
	–	–	–	–	14	NR
**R Sural – Ankle (A 7 cm, B 14 cm, C 21 cm)**						
Calf (A)	NR	NR	NR	NR	7	NR
	–	–	–	–	14	NR

ADM, abductor digiti minimi; AH, abductor hallucis; APB, abductor pollicis brevis; B Elbow, below elbow; EDB, extensor digitorum brevis; L, left; Lat, latency; Lat diff, latency difference; Min F Lat, minimum F-wave latency; Min M Lat, minimum motor latency; NR, no response; PF Amp, peak-to-peak amplitude; R, right.

Table S2. ACMG/AMP classification of the *ATP7B* c.4022-24_4025del variant.

**ACMG/AMP criterion** [1]	**Evidence supporting application**
PVS1	The deletion encompasses the canonical splice acceptor site at the intron 19/exon 20 junction and is predicted to disrupt normal splicing, potentially resulting in a frameshift and premature termination codon. (The precise transcript-level consequence was not experimentally confirmed.)*
PM2	The variant was absent from the consulted population databases, including gnomAD, the 1000 Genomes Project, and ClinVar.
PP4	The proband's clinical and biochemical findings were highly specific for wilson disease and fulfilled the EASL–ERN diagnostic criteria [2,3].
**Variant classification**	**Pathogenic variant**

* PVS1 was applied at Very Strong strength under the original ACMG/AMP framework because c.4022-24_4025del encompasses the canonical splice acceptor site and disrupts the coding sequence. Under the refined ClinGen PVS1 recommendations [4], the strength assigned to canonical splice-site variants depends on the predicted transcript consequence, the likelihood of nonsense-mediated mRNA decay, and the functional importance of the affected protein region. Because transcript analysis was not performed, the exact splicing consequence and occurrence of nonsense-mediated mRNA decay remain unconfirmed.

Table S3. Copy number variations and loss-of-heterozygosity regions identified in the proband with Wilson disease

**Cytoband**	**Chromosome Region (hg38)**	**CNVs**	**Length (bp)**	**Clinically relevant genes**
1p36.33	chr1:1-917,439	Allelic imbalance	917439	*LINC02593*
3p21.31 - p21.2	chr3:46,704,647-51,736,707	AOH	5032061	*TMIE*
9q21.11	chr9:66,715,030-67,208,576	Allelic imbalance	493547	*CNTNAP3P2*
10p11.1 - q11.1	chr10:38,489,596-39,800,000	Copy number gain*	1310405	
13q14.3 - q32.2	chr13:51,255,818-97,933,203	LOH	46677386	*ATP7B, FAM124A*
14q11.2	chr14:19,505,410-19,949,091	Copy number gain*	443682	
15q26.2	chr15:97,375,126-97,387,670	Copy number loss*	12545	*LINC02254*
16p11.2 - q11.1	chr16:29,680,484-36,800,000	LOH	7119517	*QPRT*
22q11.23 - q12.1	chr22:25,282,370-25,507,816	Allelic imbalance	225447	
Xq11.1 - q12	chrX:62,539,525-67,903,057	LOH	5363533	

*Copy number variation was classified as a variant of uncertain significance (VUS). AOH, absence of homozygosity; CNVs, copy number variations; LOH, loss of heterozygosity

Table S4. Variants of uncertain significance identified by whole-exome sequencing of the patient's husband

Variant	Gene	Chromosome position	dbSNP	Zygosity	Variant classification
NM_007114.3:c.3013-89delA	*TMF1*	Chr.3:69073420	rs201235194	Heterozygous	VUS (PM2, BP7)
NM_015541.3:c.2491+73T>G	*LRIG1*	Chr.3:66433333	rs1701165418	Heterozygous	VUS (PM2, BP7)
NM_003716.4:c.1325+20335_ 1325+20340delTGTGTG	*CADPS*	Chr.3:62611057	rs10575126	Homozygous	VUS (PM2, BP7)

Note: These variants were not confirmed due to insufficient or conflicting evidence supporting their pathogenicity.

References

1. Richards, S.; Aziz, N.; Bale, S.; Bick, D.; Das, S.; Gastier-Foster, J.; Grody, W.W.; Hegde, M.; Lyon, E.; Spector, E.; et al. Standards and Guidelines for the Interpretation of Sequence Variants: A Joint Consensus Recommendation of the American College of Medical Genetics and Genomics and the Association for Molecular Pathology. *Genet Med* **2015**, *17*, 405–424, doi:10.1038/gim.2015.30.
2. European Association for Study of Liver. EASL Clinical Practice Guidelines: Wilson's Disease. *J Hepatol* **2012**, *56*, 671–685, doi:10.1016/j.jhep.2011.11.007.
3. Socha, P.; Jańczyk, W.; Zanetto, A.; Burra, P.; Czlonkowska, A.; Debray, D.; Ferenci, P.; Merle, U.; Nicastro, E.; Poujois, A.; et al. EASL-ERN Clinical Practice Guidelines on Wilson's Disease. *Journal of Hepatology* **2025**, *82*, 690–728, doi:10.1016/j.jhep.2024.11.007.
4. Abou Tayoun, A.N.; Pesaran, T.; DiStefano, M.T.; Oza, A.; Rehm, H.L.; Biesecker, L.G.; Harrison, S.M.; ClinGen Sequence Variant Interpretation Working Group (ClinGen SVI) Recommendations for Interpreting the Loss of Function PVS1 ACMG/AMP Variant Criterion. *Hum Mutat* **2018**, *39*, 1517–1524, doi:10.1002/humu.23626.
